# Mechanical Behavior of GFRP Connection Using FRTP Rivets

**DOI:** 10.3390/ma14010007

**Published:** 2020-12-22

**Authors:** Takayoshi Matsui, Yoshiyuki Matsushita, Yukihiro Matsumoto

**Affiliations:** 1 Department of Architecture and Civil Engineering, Toyohashi University of Technology, Aichi, Toyohashi 441-8580, Japan; y-matsum@ace.tut.ac.jp; 2 IO INDUSTRY CO., Ltd., Kosai, Shizuoka 431-0302, Japan; matsushitay@io-industry.co.jp

**Keywords:** GFRP, FRTP, rivet, connection

## Abstract

In recent years, the application of fiber-reinforced plastics (FRPs) as structural members has been promoted. Metallic bolts and rivets are often used for the connection of FRP structures, but there are some problems caused by corrosion and stress concentration at the bearing position. Fiber-reinforced thermoplastics (FRTPs) have attracted attention in composite material fields because they can be remolded by heating and manufactured with excellent speed compared with thermosetting plastics. In this paper, we propose and evaluate the connection method using rivets produced of FRTPs for FRP members. It was confirmed through material tests that an FRTP rivet provides stable tensile, shear, and bending strength. Then, it was clarified that non-clearance connection could be achieved by the proposed connection method, so initial sliding was not observed, and connection strength linearly increased as the number of FRTP rivets increased through the double-lapped tensile shear tests. Furthermore, the joint strength of the beam using FRTP rivets could be calculated with high accuracy using the method for bolt joints in steel structures through a four-point beam bending test.

## 1. Introduction

Fiber-reinforced plastics (FRPs) are used for repairing and reinforcing structures because they have a high strength-to-weight ratio and corrosion resistance. In recent years, the application of FRPs as structural members, e.g., in pedestrian bridges, buildings, and large roofs, has been promoted [1]. Bai and Keller [2] introduced an FRP pedestrian bridge built in 2005 and investigated its dynamic response behavior. They suggested that the connection method affects structural dynamic behavior. Votsis et al. [3] investigated the structural behavior of a novel type of FRP bridge—the Aberfeldy footbridges—and they evaluated their dynamic properties by experiment and the finite element method to clarify the long-term performance of FRP bridges. Evernden and Mottram [4] introduced FRP buildings and their manufacturing process to develop FRP buildings in the United Kingdom. Yang et al. [5] proposed new space frame structures with a grass fiber-reinforced plastic (GFRP) connection method, and the method of structural design and modeling for finite element analysis was clarified. Matsumoto and Yonemaru [6] investigated mechanical performance of a CFRP roof truss member under long-term loading conditions, and it was confirmed that properties were not varied even if specimens were exposed outside. In order to use FRPs as structural members, it is necessary to study the connection method. Several methods have been proposed for connecting FRP members, such as mechanical joints, adhesively bonded joints, and composites of these. Coelho and Mottram [7] summarized the bolted connection and its strength, and they suggested that the connection of pultruded FRP should be established by considering the material characteristics, such as orthotropic material properties and the estimation of several failure modes. Ueda et al. [8] proposed a new connection method using a carbon fiber-reinforced thermoplastics (CFRTP) rod to joint CFRP plates like a rivet. They clarified the shear strength of CFRTP rod with 5.2 mm diameter as 2.5–3.6 kN, and demonstrated that the specific joint strength can be effectively increased. Ascione et al. [9] proposed adhesively bonded GFRP beam-column connections with an improved connection by angle member and stiffener, and experimentally investigated the connection strength. They concluded that the fully bonded connection could provide rigid and higher connection strength with cohesive failure. However, strength variation and stability was not evaluated depending on bonding condition and material imperfection. In the case of mechanical joints, metallic bolts and rivets are often used, but the corrosion resistance of FRP member is not fully utilized because these joints are degraded by corrosion. Furthermore, a bolted connection lacks initial stiffness because frictional resistance cannot be performed. Connection strength may also decrease due to unavoidable non-uniform clearances between base member and bolt/rivet shank because an FRP member cannot redistribute bearing stress by plastic deformation. Marra et al. [10] clarified un-uniform load distribution in multi-bolt joints because of bolt-hole clearance and bolt position by finite element analysis, and they evaluated the stress distribution coefficients. However, an improved method was not suggested. Matsumoto et al. [11] reported that the diameter ratio of the bolt and bolt hole greatly affects bearing strength, and bearing strength can be improved by decreasing the diameter of the bolt hole to close a clearance.

On the other hand, fiber-reinforced thermoplastics (FRTPs), which are FRPs that use thermoplastic resins, have attracted attention in composite material fields. FRTPs can be remolded by heating, so they have the possibility of secondary processing, recycling, and reuse in addition to FRPs. Moreover, FRTPs could reduce costs because they can be manufactured with excellent speed by injection molding or press molding compared with thermosetting plastics. Yeong et al. [12] demonstrated the manufacture of a CFRTP and GFRTP coupon, and they tested them under tension, bending, and indentation loads. They suggested that the manufacturing process using FRTP could reduce molding costs, and showed comparable strength and elastic modulus to conventional CFRP and GFRP. To develop and apply FRTPs to engineering fields, research for the evaluation of mechanical characteristics by Doan [13] and Cao [14] was carried out, and manufacturing processes were also evaluated in recent years by Fan [15] and Rodonò [16].

On the basis of this background, we studied the connection methods to take advantage of the features of FRP materials and structures. In this paper, we propose a connection method using FRTP rivets to provide a solution for problems caused by corrosion and clearance, and evaluate the connection strength and mechanical behavior through material tests and structural experiments.

## 2. Fiber-Reinforced Thermoplastic (FRTP) Rivet and Its Connection Method

**Figure 1:**
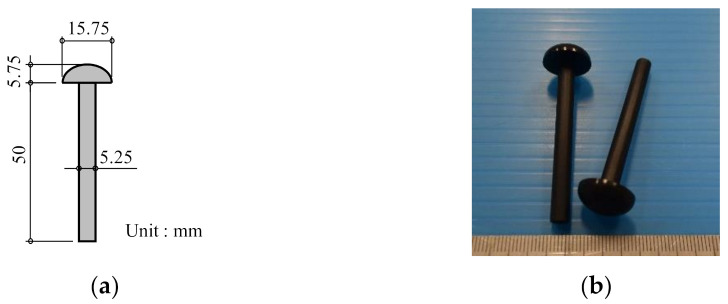
Fiber-reinforced thermoplastic (FRTP) rivet. (**a**) Geometry of FRTP rivet; (**b**) image of FRTP rivets.

Figure 1 shows the geometry and image of the FRTP rivets used in this study. The FRTP rivets were made of polyamide 6 (PA6) with 50 wt.% glass fiber (Durethan BKV50HEF 900,116 DUS022, Tokyo, Japan) by injection molding. The length of the glass fiber was approximately 0.35 mm. Table 1 shows the properties of the FRTP material.

**Figure 2:**
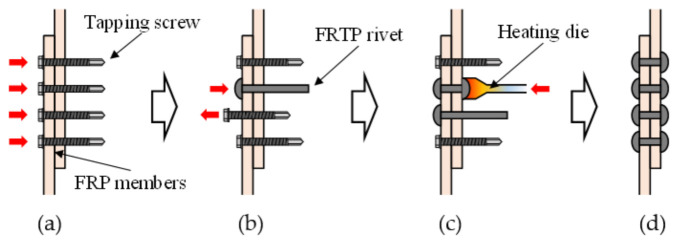
Connection method. (**a**) Tentative connection by tapping screw to produce holes and fix fiber-reinforced plastic (FRP) members; (**b**) ejecting tapping screw and inserting FRTP rivets; (**c**) thermoforming rivet head; (**d**) completed FRTP connection.

We evaluated the connection method using tapping screws that do not require holes to insert a tapping screw for FRP members [17]. The initial stiffness of the connection increased, and stress concentration was reduced because there was no clearance between FRP base member and tapping screw. However, the connection using the tapping screw lacked pull-out and fatigue strength because it did not contain a nut at the drilling side. To improve the non-clearance connection, the connection method using FRTP rivets and tentative tapping screws was proposed to provide higher initial connection stiffness and minimize preparation for mechanical connection. Figure 2 shows the connection method using FRTP rivets. First, a tentative connection by tapping screws to produce holes and fix FRP members was performed (Figure 2a). The nominal outer diameter of the tapping screw was 5.5 mm. Second, tapping screws were ejected while fixing FRP members, and FRTP rivets were inserted (Figure 2b). Third, the rivet head was thermoformed using a heating die (Figure 2c). Processes (b) and (c) were applied to all FRTP rivets one by one. Lastly, the FRTP connection could be made as shown in Figure 2d. In addition, a tentative connection by tapping screws does not necessarily have to be drilled for all rivet positions. In this case, other rivet holes could be drilled with a drilling machine in the same way as in bolt-hole preparation.

## 3. Material Tests

### 3.1. Tensile Test

**Figure 3:**
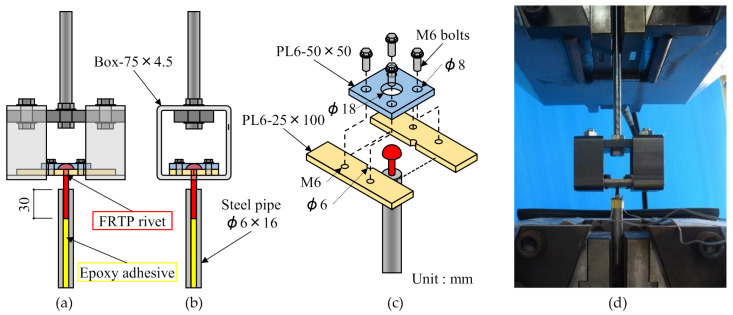
Material tensile test method: (**a**) Front view; (**b**) side view; (**c**) detail of testing parts; (**d**) experiment setup.

Figure 3 shows the material tensile test method and setup. Total number of specimens for material tensile test was 5. Tensile force was applied to the FRTP rivet through the bottom steel pipe and top testing frame because it was difficult to directly apply tensile force to the FRTP rivet. The FRTP rivet was fixed to the steel pipe using epoxy adhesive with 30 mm length for chucking to the tensile testing machine. The shank of the FRTP rivet was then sandwiched between two half-notched steel plates and fixed with another steel plate and bolts. Then, it was hooked on a test frame combined with square steel pipe.

**Figure 4:**
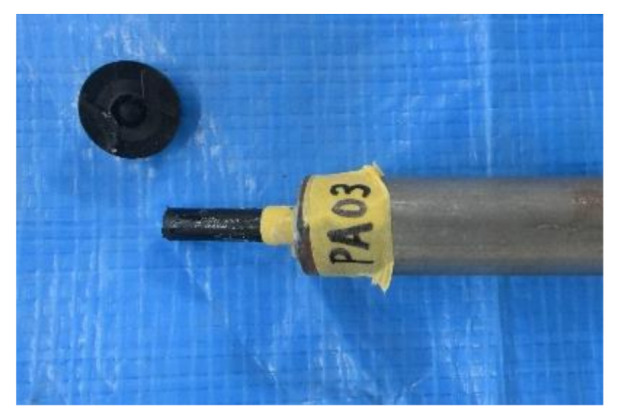
Failure mode of FRTP rivet under tensile loading.

**Figure 5:**
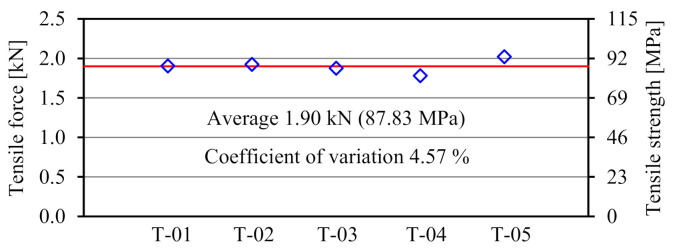
Material tensile test results.

Figure 4 shows the failure mode of the FRTP rivet under tensile loading. Material tensile breaking occurred at the corner between rivet head and shank in all specimens. Figure 5 shows the tensile forces, average value, and coefficient of variation obtained by the material tensile test. The tensile strength of the right vertical axis was obtained by dividing the tensile force of the left vertical axis by the cross-section area of the rivet shank with an outer diameter of 5.25 mm. The tensile strength of the FRTP rivet was evaluated to be approximately 69% of the nominal tensile strength of the material at wet condition, as shown in Table 1. This is because stress concentration occurred due to rapid cross-sectional changing at the corner between rivet head and shank. Stable tensile strength of the FRTP rivet is demonstrated because of the small coefficient of variation.

### 3.2. Tensile Shear Test

**Figure 6:**
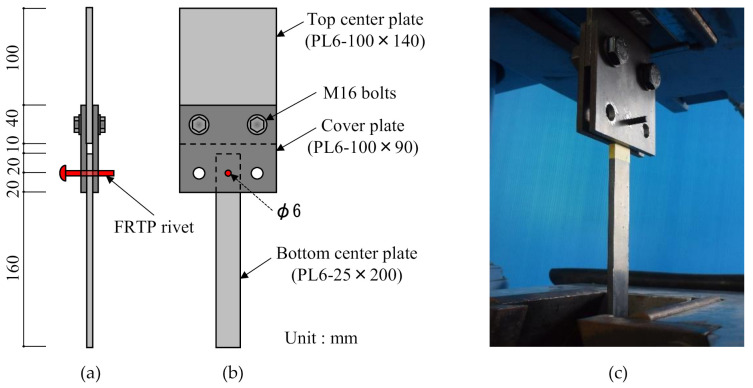
Material shear test method: (**a**) Front view; (**b**) side view; (**c**) experiment setup.

Figure 6 shows the material tensile shear test method and setup. Total number of specimens for material tensile shear test was 10. Shear force was applied to the FRTP rivet through steel plates. The top center plate and cover plates were fixed by high-strength bolts. The FRTP rivet was inserted into a 6 mm hole of cover plates and bottom center plate to apply shear load without bending.

**Figure 7:**
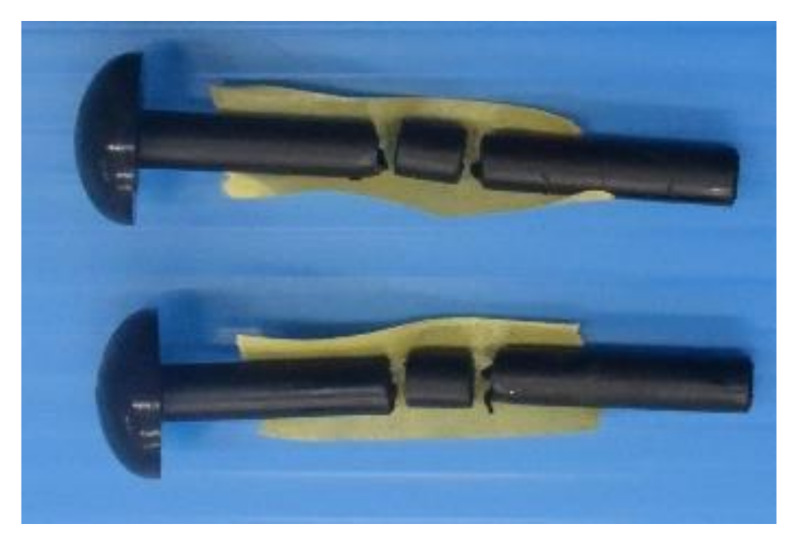
Failure mode of FRTP rivet under shear loading.

**Figure 8:**
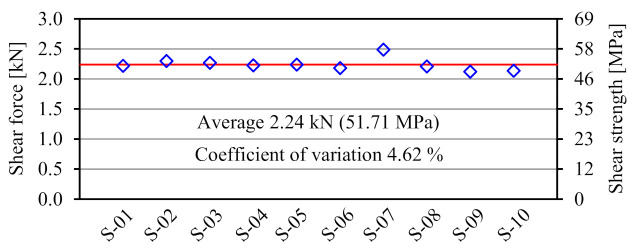
Material tensile shear test results.

Figure 7 shows the failure mode of FRTP rivet under shear loading. Material shear breaking occurred at the boundaries between steel plates in all specimens. Figure 8 shows the shear forces, average value, and coefficient of variation obtained by the material tensile shear test. The shear strength of the right vertical axis was obtained by dividing the shear force of the left vertical axis by twice the cross-section area of the rivet shank, because shear force was applied to two cross-sections of the rivet shank. The stable shear strength of the FRTP rivet is demonstrated because of the small coefficient of variation.

### 3.3. Bending Test

**Figure 9:**
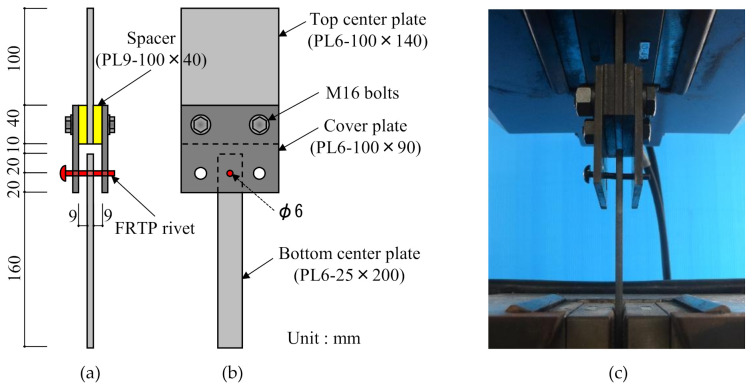
Material bending test method: (**a**) Front view; (**b**) side view; (**c**) experiment setup.

Figure 9 shows the material bending test method and setup. Total number of specimens for the material bending test was 10. Bending force was applied to the FRTP rivet by using the longitudinally extended test frame of the material shear test by 9 mm thickness spacers inserted between cover plates and top center plate.

**Figure 10:**
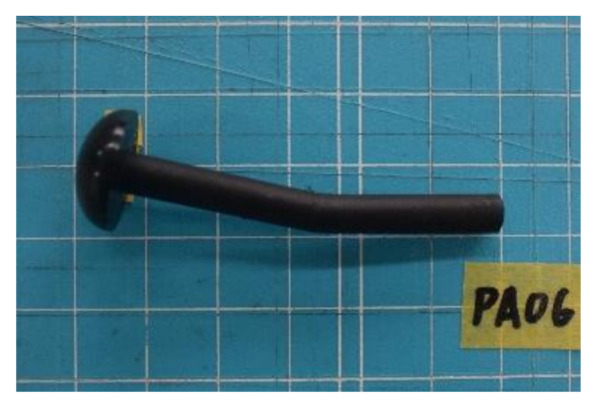
Failure mode of FRTP rivet under bending.

**Figure 11:**
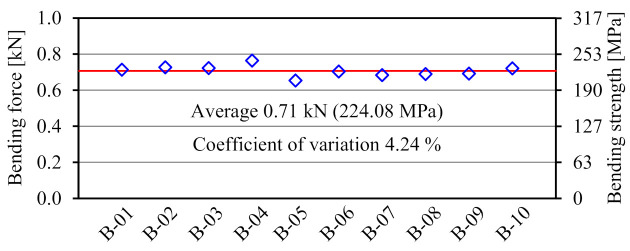
Material bending test results.

Figure 10 shows the failure mode of the FRTP rivet under bending loading. Material bending breaking occurred at the center of rivet shank all specimens. Figure 11 shows bending forces, average value, and coefficient of variation obtained by the material bending test. The bending strength of right vertical axis $\sigma_{R}$ was calculated by the bending force of left vertical axis $F_{R}$ from the following equation.

(1) $$\sigma_{R}=\frac{F_{R}l_{B}}{2Z_{R}}$$

where $l_{B}$ is the distance between steel plates ($l_{B}$ = 9 mm), and $Z_{R}$ is the section modulus of the rivet shank. The bending strength of the FRTP rivet was between the nominal bending strengths of material at dry and wet conditions. In addition, a small coefficient of variation was observed, so the stable bending strength of the FRTP rivet could be demonstrated.

## 4. Connection Strength under Tensile Shear Loading

**Figure 12:**
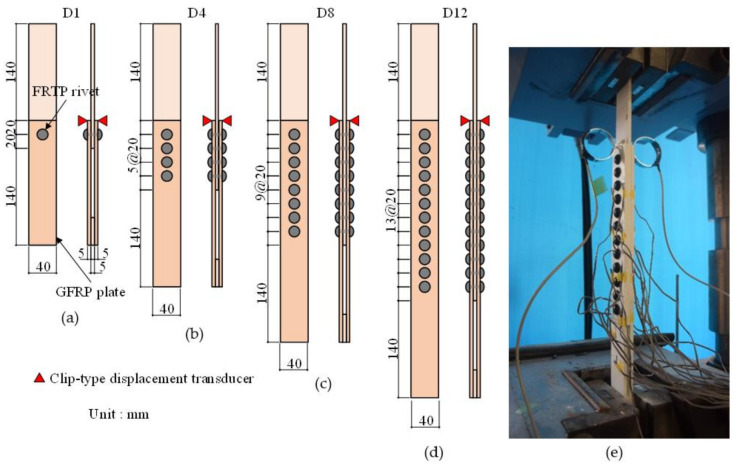
Connection test specimens: (**a**) Single rivet; (**b**) four rivets; (**c**) eight rivets: (**d**) 12 rivets; (**e**) experiment setup (12-rivet specimen).

This section discusses the connection strength using single or multiple FRTP rivets for GFRP plates through double-lapped tensile shear tests. Figure 12 shows the connection test specimens and setup. Double-lapped GFRP plates with 40 mm width connected by FRTP rivets were used. GFRP plates were made by pultrusion using unsaturated polyester resin and glass fiber (PLALLOY^TM^ by AGC Matex Co., Ltd., Kanagawa, Japan); the glass roving layer (approx. 4.5 mm) was sandwiched between the continuous strand mat layers (approximately 0.25 mm). Table 2 shows the mechanical properties of the GFRP plate. The number of specimens for the connection test was 5 for each connection type depending on the number of used rivets: 1, 4, 8, and 12. The rivets were arranged low at intervals of 20 mm. Specimen names were D1, D4, D8, and D12 according to the number of rivets used.

All specimens were monotonically tested using a tensile test machine. Two clip-type displacement transducers were mounted on both sides of the specimen to measure the relative displacement of the connection, as shown in Figure 12.

**Figure 13:**
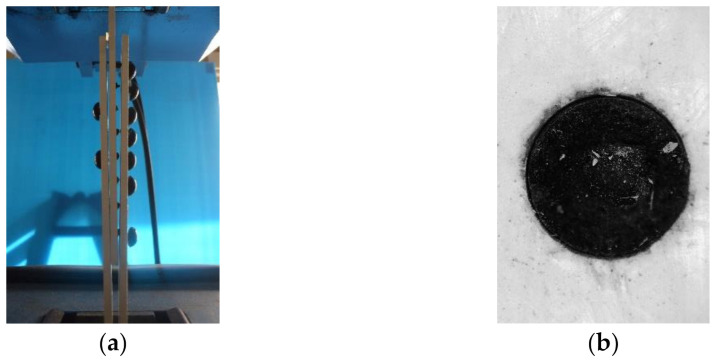
Typical failure mode. (**a**) Failure mode of eight-rivet specimen; (**b**) close-up image around FRTP rivet.

**Figure 14:**
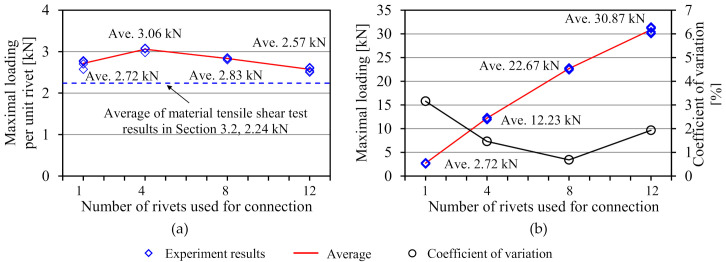
Experiment result. (**a**) Maximal loading; (**b**) maximal loading per unit rivet.

**Figure 15:**
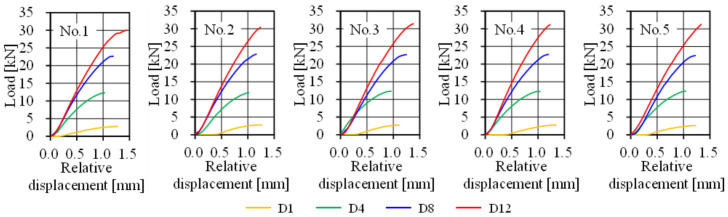
Load-relative displacement relations.

As a result of the connection test, the shear failure of the FRTP rivets was obtained in all specimens, as shown in Figure 13a. Figure 14a shows the maximal loading, average values, and standard deviation obtained by the connection test. Average strength obtained by the D1 specimens was 2.72 kN, which was 21% higher than the average of the material test result. This is because a slight bending moment was applied due to the clearance between cover plate and rivet in the material shear test, but only shear force could be applied in the connection test by the non-clearance connection method shown in Figure 2. Figure 13b shows a close-up image of around the FRTP rivet of the broken specimen. Since the nominal outer diameter of the tapping screw was 5.5 mm, the clearance between FRTP rivet and GFRP plate was theoretically 0.25 mm. In fact, Figure 13b confirms that the clearance was very small. The connection using FRTP rivets could provide quite stable strength because of the small coefficient of variation regardless of the number of rivets. Figure 14b shows the maximal loading per unit rivet. Maximal loading per unit rivet was calculated by the maximal loading shown in Figure 14a divided by the number of rivets used for the connection. Even if the number of rivets increased, the connection strength per unit rivet was almost the same. Moreover, the connection strength per unit rivets was higher than the shear strength of material test was. Therefore, connection strength using FRTP rivets linearly increased as the number of rivets increased. Figure 15 shows the load-relative displacement relations. Initial sliding was not observed in all specimens using multiple rivets because non-clearance could be achieved, as shown in Figure 13b.

## 5. Mechanical Behavior of Beam Joint under Bending

**Figure 16:**
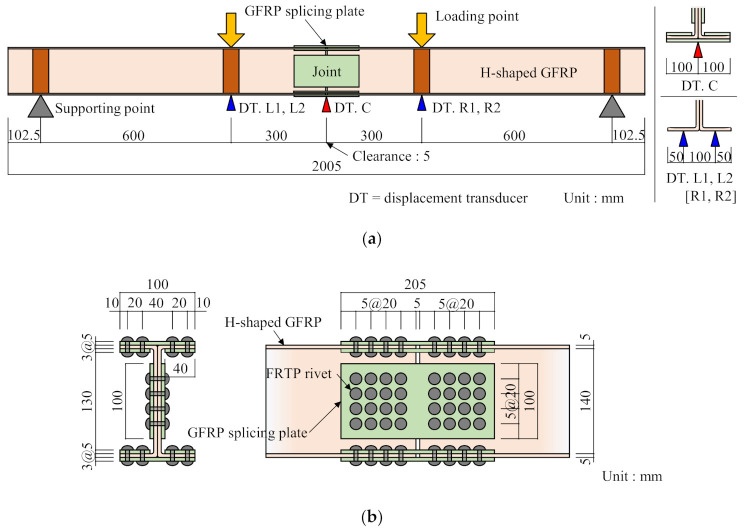
Bending test specimen. (**a**) Specimen overview; (**b**) joint detail.

**Figure 17:**
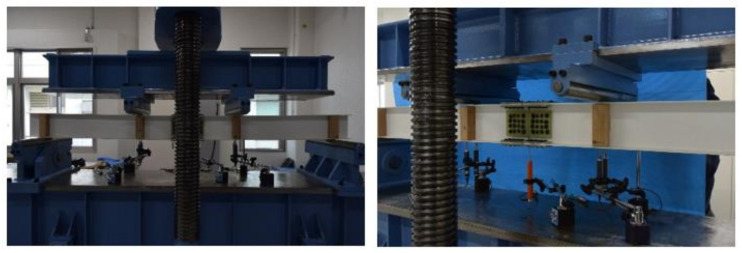
Experiment setup.

This section discusses the strength of the beam joint using FRTP rivets for a GFRP beam through a four-point bending test. Figure 16 and Figure 17 show bending test specimen and setup, respectively. The specimen was produced with longitudinally jointed two H-shaped GFRP beams with a 5 mm gap using FRTP rivets through GFRP splice plates. The H-shaped GFRP beam member consisted of two pultruded GFRP channel-shaped members that were adhesively bonded back-to-back by epoxy adhesive. The mechanical properties of the GFRP channel-shaped member are shown in Table 2. The GFRP splicing plates were molded by vacuum-assisted resin transfer molding using two-directional glass woven fabric (ERW580-554A) and epoxy resin.

Figure 16b shows the joint details. The number of rivets in width, height, and longitudinal direction were four, four, and eight, respectively. Thus, the total number of rivets used for joining was 96. The total number of rivets was determined so that the rivet fracture preceded the beam member fracture and buckling to evaluate the strength of the FRTP rivet connection. The bending test was performed on only one specimen, but bending test behaviors were expected to be stable because connection strength using FRTP rivets was stable, as shown in Section 4.

**Figure 18:**
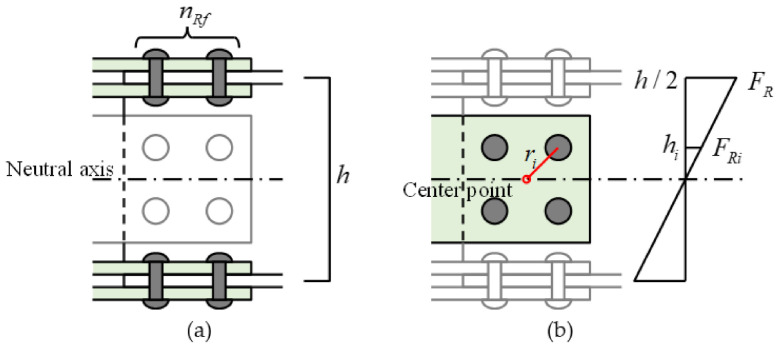
Calculation of maximal loading at the joint. (**a**) Flange; (**b**) web.

Maximal loading at the joint was calculated by multiplying rivet strength and distance from the neutral axis or the center point of rotation with the same method as that for bolt joints in steel structures, as shown in Figure 18. Bending moment resisted by flange rivets $M_{f}$ was calculated by the following equation.

(2) $$M_{f}=n_{Rf}\cdot F_{R}\cdot h$$

where $n_{Rf}$ is the number of FRTP rivets in upper/lower flange of one side of beam member $F_{R}$ is the shear force of the FRTP rivets, which was obtained from D1 specimens shown in Figure 12 of the connection test; and h is distance between the center of the thickness of the upper and lower flanges. In this study, $n_{Rf}=16$, $F_{R}=2.72$ kN, and h=145 mm. Bending moment resisted by web rivets $M_{w}$ was calculated by the following equation.

(3) $$M_{w}=\sum r_{i}\cdot F_{Ri}=\sum r_{i}\cdot \frac{h_{i}}{h/2}\cdot F_{R}$$

where $r_{i}$ is distance from the center of gravity of web joint to one rivet; and $F_{Ri}$ is the shear force resisted by the rivet, which was calculated by multiplying the shear force of FRTP rivets $F_{R}$ by the ratio of the vertical distance of rivet $h_{i}$ to the vertical distance of center of flange h/2 based on the beam neutral axis. Therefore, maximal loading at the joint $P_{R}$ was calculated using the bending moments obtained from the Equations (2) and (3) by the following equation.

(4) $$P_{R}=2\frac{M_{f}+M_{w}}{l}$$

where l is distance from loading point to supporting point of the specimen (l=600 mm). As a result of the calculation, maximal loading at the joint could be estimated as 22.35 kN.

The specimen was monotonically tested using a compression test machine. Displacement transducers were mounted onto the middle of the span and the loading points of the specimen to measure the displacement of the specimen, as shown in Figure 16a.

**Figure 19:**
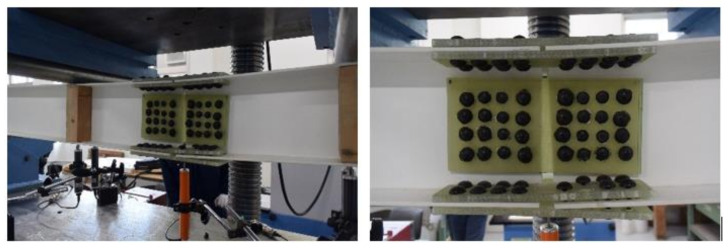
Failure mode.

**Figure 20:**
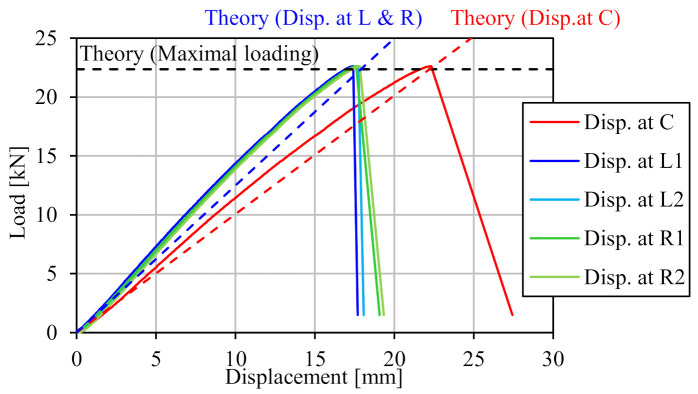
Load-displacement relations.

Figure 19 shows the failure mode of the bending test specimen. Rivets were almost broken, with shear failure mode on the right side of the web and the bottom flange, and the failure position was the surface between web and splice plate. This failure mode was as expected because shearing force acting on the rivets reached the maximum at the top and bottom of the beam. Figure 20 shows the load-displacement relations. The load rapidly decreased due to the rivets breaking after reaching maximal loading. The maximal loading obtained by bending test was 22.61 kN, which had a 1.2% error from theoretical value $P_{R}$. Thus, it is demonstrated that joint strength using FRTP rivets can be calculated with high accuracy from the shear strength of a rivet and the distance from the center using the method for bolt joints in steel structures. Maximal displacement at the middle of span obtained by bending test was 22.26 mm, and the average of the displacements at the loading points of the specimen obtained by bending test was 17.60 mm.

**Figure 21:**
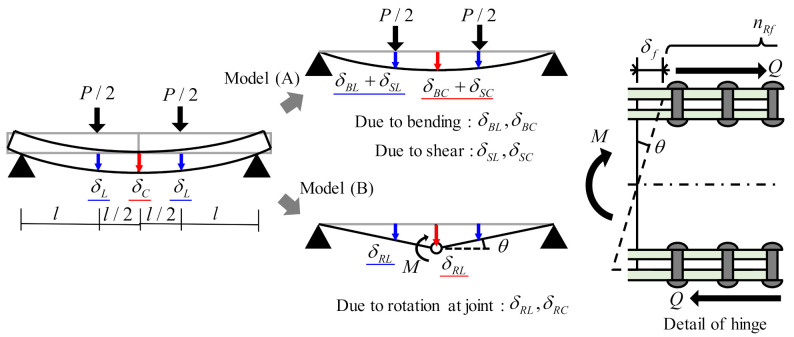
Displacement components of FRTP rivet connection.

In this study, we considered three displacement components to evaluate deformation, i.e., the bending and shear deformations obtained from fundamental beam theory, and rotation at the joint as shown in Figure 21. Displacements due to bending, $\delta_{BC}$ and $\delta_{BL}$ were calculated by the following equations. Subscript *C*, middle of span; subscript *L*, loading points.

(5) $$\delta_{BC}=\frac{23Pl^{3}}{48EI}$$

(6) $$\delta_{BL}=\frac{5Pl^{3}}{12EI}$$

where P is the applied load, E is the longitudinal elastic modulus of the H-shaped GFRP beam shown in Table 2, and I is the moment of inertia of the H-shaped GFRP beam. Displacements due to shear stress $\delta_{SC}$ and $\delta_{SL}$ were calculated by the following equation.

(7) $$\delta_{SC}=\delta_{SL}=\frac{\kappa Pl}{2GA}$$

where κ is the shear correction factor, which is the ratio of the web cross-sectional area to the total cross-sectional area of the H-shaped GFRP beam; G is the shear modulus of the H-shaped GFRP beam; and A is the cross-sectional area of the H-shaped GFRP beam. Figure 21 shows a model in which the joint of the beam was an elastic hinge to obtain the displacement due to rotation at the joint. Shear force resisted by upper/lower flange rivets Q was calculated from bending moment applied at the joint of beam M by the following equation.

(8) $$Q=\frac{M}{h}=\frac{Pl}{2h}$$

Longitudinal displacement of flange plate by rivet deformation of $\delta_{f}$ was calculated by the following equation.

(9) $$\delta_{f}=\frac{Q}{n_{Rf}K_{R}}=\frac{Pl}{2n_{Rf}hK_{R}}$$

where $K_{R}$ is the shear stiffness of the FRTP rivet; and $K_{R}=4.12$ kN/mm, which was obtained from the D1 specimens shown in Figure 12. Angle of rotation at the joint, θ was calculated by the following equation.

(10) $$\theta =\frac{2\delta_{f}}{h}=\frac{Pl}{n_{Rf}h^{2}K_{R}}$$

Displacements due to rotation at the joint, $\delta_{RC}$ and $\delta_{RL}$ were calculated by multiplying the angle of beam rotation by the distance from the support point as per the following equations.

(11) $$\delta_{RC}=\theta \cdot \frac{3}{2}l=\frac{3Pl^{2}}{2n_{Rf}h^{2}K_{R}}$$

(12) $$\delta_{RL}=\theta \cdot l=\frac{Pl^{2}}{n_{Rf}h^{2}K_{R}}$$

On this basis, displacement at the middle of span and the loading point of the specimen was calculated from the sum of the deformation components as per the following equations.

(13) $$\delta_{C}=\delta_{BC}+\delta_{SC}+\delta_{RC}$$

(14) $$\delta_{L}=\delta_{BL}+\delta_{SL}+\delta_{RL}$$

Theoretical displacements obtained by the Equations (13) and (14) are also represented in Figure 18. They were in good agreement with the displacements obtained by bending test at maximal loading; errors were 1.1% at the middle of span and 2.9% at the loading points. Therefore, displacement at maximal loading could be accurately evaluated by considering displacement due to rotation at the joint in addition to displacement due to bending and shear. Displacement during loading could not be perfectly simulated because the obtained displacement from the bending test increased nonlinearly with the load. However, theoretical displacement at the middle of span could be simulated with 15% error the experimental displacement.

## 6. Conclusions

This paper proposed a connection method of FRP members using FRTP rivets and evaluated the fundamental properties of FRTP rivets through material tests. Connection behavior was also evaluated through the double-lapped tensile shear tests and a four-point bending test of the beam. As a result, the following conclusions were reached.

(1) FRTP rivets provide stable tensile, shear, and bending strength within 4.7% of the coefficient of variation.
(2) Connection strength using FRTP rivets linearly increases as the number of rivets increases.
(3) Initial sliding was not observed at the FRTP rivet connection because non-clearance could be achieved by the proposed connection method.
(4) Beam joint strength using FRTP rivets could be calculated with high accuracy within 1.2% error from the shear strength of a rivet like a bolt joint in steel structure.
(5) Beam displacement using FRTP rivets at maximal loading could be accurately evaluated within 2.9% error by considering displacement due to rotation at the joint in addition to displacement due to bending and shear deflection.

On the basis of this research, GFRP members can be connected with stable loading capacity, and deflections can be evaluated with high accuracy by using FRTP rivet even if bolt holes are not prepared. The connection method in this paper could also reduce construction time and improve the material machining process because onsite drilling can be applied by tapping screws.

## Figures and Tables

**Table 1:** Properties of FRTP material.

Property	Dry Condition	Wet Condition	Testing Method
Tensile Strength (MPa)	205	128	ISO527-1,-2
Elastic Modules (GPa)	16.2	9.5	ISO527-1,-2
Bending Strength (MPa)	320	203	ISO178A
Melting Point (°C)	220		ISO11357-1,-3
Density	1.57		ISO1183

**Table 2:** Mechanical properties of grass fiber-reinforced plastic (GFRP).

Property	Value	Testing Method
Fiber content (wt.%)	53	JIS K 7165
Longitudinal tensile strength (MPa)	411	JIS K 7165
Longitudinal elastic modulus (GPa)	28	JIS K 7052
